# F66. DO CLINICAL VARIABLES DURING THE EARLY ILLNESS PERIOD PREDICT THE COGNITIVE COURSE IN EARLY-ONSET SCHIZOPHRENIA?

**DOI:** 10.1093/schbul/sby017.597

**Published:** 2018-04-01

**Authors:** Charlotte Teigset, Christine Mohn, Bjorn Rund

**Affiliations:** University of Oslo

## Abstract

**Background:**

Early-onset schizophrenia (EOS) affects approximately 5% of the schizophrenia population, and reflects increased disease severity, with a worse clinical course and outcome. Because of extensive brain maturation in the adolescence, the EOS patients provide unique neurodevelopmental data that may contribute to a better understanding of schizophrenia at all ages. Cognitive dysfunction is a central feature of schizophrenia, and is assumed to be more pronounced in EOS than in later onset illness. Previously, we have reported a deteriorated, but stable cognitive course in EOS,1 and examined the relationship between cognition and symptoms (submitted).2 While both cognition and clinical variables have been subject to comprehensive research in schizophrenia, the interaction between the two has gained less attention, especially in EOS. An essential question now, is to what extent the longitudinal course of cognition is influenced by clinical variables in the early illness period.

**Methods:**

Thirty-one EOS patients and 73 controls (age 12–18) were assessed on clinical variables at baseline (PANSS, duration of untreated psychosis [DUP], hospitalizations, suicide attempts and remission). Neuropsychological assessments with the MATRICS Consensus Cognitive Battery (MCCB) were conducted at baseline, after both one and two years, and composite scores of total performance were calculated. The analyses were performed with a linear mixed model.

**Results:**

In the present study, both PANSS-general and suicide attempt history at baseline were identified as risk factors of longitudinal cognitive function. We did not detect a relationship between DUP, remission, positive/negative symptoms and hospitalizations on the one hand, and long-term cognition on the other.

**Discussion:**

Some baseline characteristics (psychotic symptoms, DUP, remission and hospitalization) had no influence on cognition within the first two years of illness. In contrast, we found that a higher amount of general symptoms (PANSS) and a history of suicide attempts at baseline significantly predicted a deteriorated longitudinal composite score in EOS. This may imply that cognitive deterioration is influenced by a strong affective response to the illness, rather than a result of irrational or psychotic symptoms in and of themselves. Our findings indicate that higher scores of general symptoms, as well as suicide attempt history, predict a deteriorated cognitive course, and should be subject to specific attention in the evaluation and treatment of patients with early-onset psychosis.

**References:**

1. Juuhl-Langseth, M., Holmen, A., Thormodsen, R., Oie, M., & Rund, B. R. (2014). Relative stability of neurocognitive deficits in early onset schizophrenia spectrum patients. Schizophrenia Research, 156(2–3), 241–247. doi:10.1016/j.schres.2014.04.014

2. Teigset, C., Mohn, C., Brunborg, C., Holmen, A., Juuhl-Langseth, M., Rund, B. (2018). Do clinical characteristics predict the cognitive course in early-onset schizophrenia-spectrum disorders?

